# Editorial - Using death investigation data to better understand the overdose crisis

**DOI:** 10.24095/hpcdp.44.3.01

**Published:** 2024-03

**Authors:** Amanda VanSteelandt, Jenny Rotondo

**Affiliations:** 1 Public Health Agency of Canada, Ottawa, Ontario, Canada

## Introduction

In response to the overdose (or acute drug toxicity) crisis, the Government of Canada committed to a public health response supported by a strong evidence base.^1^ Information regarding the people affected, their exposure to risk factors, opportunities to connect them to supports, the circumstances of their death and the substances involved is critical to effectively support policy decisions and identify and prioritize potential interventions to reduce drug toxicity events. 

The collection of articles in this theme series of the journal presents results from a national chart review study of the death investigation files of people who died of acute toxicity in Canada between 2016 and 2017. This study endeavoured to better understand the characteristics of the people who died, the circumstances of their deaths and the substances involved. While information about the study and some of its findings have previously been published,^2^,^3^ we would like to share more about how the study came to be, the people involved and the value of collaborative efforts between coroners, medical examiners and public health practitioners.

Work on the national chart review study began in 2018 to complement the national surveillance system of apparent opioid- and stimulant-related related acute toxicity deaths.^4^ Both activities aimed to address the lack of comparable national data on acute toxicity mortality using death investigation data, and neither could have proceeded without the collaboration of chief coroners, chief medical examiners and their offices across provinces and territories. The mandate of coroners and medical examiners is to establish the cause and manner of non-natural deaths. During the course of their investigations, they collect a variety of information, including interviews with family members, witnesses and medical practitioners; descriptions of the scene of the death; medical and law enforcement records; and autopsy and toxicology reports. The chart review study brought these sources of information together in one dataset, which is being analyzed by multiple research teams to answer key questions about the overdose crisis. As the articles in this series demonstrate, these data can be a rich source of public health information not available elsewhere from which we can learn more about the groups of people most affected and discover ways to tailor policies and programs to better meet their needs.

Though the study was funded and coordinated by staff at the Public Health Agency of Canada (PHAC), the co-investigator team leading the vision and design of the study was made up of multidisciplinary members from many organizations. The team was assembled to represent voices from across Canada with a variety of expertise, including lived experience of substance use, death investigations, harm reduction programs, addictions counselling, toxicology, health equity, Indigenous health research, geomatics, health care and public health. Decisions about how to analyze the dataset were informed by the co-investigator team in combination with priorities identified by stakeholder groups that included people with lived and living experience of substance use and government policy leads. 

The collection of articles in this theme series focusses on specific subpopulations of people who died accidentally of acute toxicity from 2016 to 2017, including youth^5^ and older adults.^6^ A future issue will include articles on additional populations. The articles share common threads examining the prevalence of characteristics related to social and medical history, circumstances of death and substances involved for each of these groups. The overdose crisis has affected people of all walks of life, and these articles demonstrate the diversity of the circumstances surrounding deaths and the importance of taking an intersectional approach when assessing how factors affect risk across populations.^7^

An important challenge to applying an intersectional approach to analyses is identifying subpopulations within the dataset. As the mandate of coroners and medical examiners is to establish the cause and manner of non-natural deaths, we found that most files were missing information relevant for public health purposes, particularly regarding socioeconomic and social identity characteristics. Therefore, the results of the national chart review study represent the *minimum* prevalence of characteristics among the people who died. 

The process of identifying what information was available in death investigation files and comparing this across provinces and territories has supported collaborative efforts between coroners, medical examiners and public health practitioners to improve the data available and the knowledge base on acute toxicity deaths. Some of these discussions are centred around how to better identify additional subpopulations and evaluate their risk of acute toxicity, including subpopulations based on self-identity (e.g. race, ethnicity, distinctions-based Indigenous status, gender and sexual orientation) and life experiences (e.g. involvement with correctional systems, foster care, occupational groups and potentially traumatic experiences), which were often unavailable in death investigation files.

The national chart review study provides a snapshot of the overdose crisis in its early years. While it serves as a national baseline for future research, some of the patterns observed in the theme series articles may have changed, as the drug supply has become increasingly toxic, harm reduction and treatment options have expanded and the social circumstances of Canadians have changed since 2016 and 2017.^8^,^9^ Even with the support of the coroner and medical examiner offices, planning and data collection for the national chart review study took over four years. Though this was partly due to the COVID-19 pandemic, data abstraction from physical files is time-intensive, and many of the relationships, processes and agreements necessary to proceed with this project did not exist at the outset. 

Hopefully, the groundwork laid by this study will support similar activities across provinces and territories, improvements to Statistics Canada’s Canadian Coroner and Medical Examiner Database,^10^ and the work of the Chief Coroner, Chief Medical Examiners and Public Health Collaborative. The latter group comprises members from PHAC, all 13 Canadian chief coroner and chief medical examiner offices and Statistics Canada, with a mandate to support the development of common approaches to death investigations and data infrastructure requirements.

Timely and comparable data are essential to the development of a robust evidence base that public health professionals can use to address the evolving national overdose crisis and its drivers. Key to developing this evidence base is fostering collaborative relationships between multidisciplinary teams, including the public health sector and the coroner and medical examiner community.

## Acknowledgements

We would like to acknowledge our collaborators at the offices of chief coroners and chief medical examiners across Canada for providing access to their death investigation files. We are deeply thankful to our co-investigators on the National Chart Review Study of Substance-Related Acute Toxicity Deaths for their continued guidance and participation in the project over the last five years: Brandi Abele, Songul Bozat-Emre, Matthew Bowes, Jessica Halverson, Dirk Huyer, Beth Jackson, Graham Jones, Jennifer Leason, Regan Murray, Erin Rees and Emily Schleihauf. We are grateful for the contributions of people with lived and living experience of substance use who have supported the chart review study at multiple stages.

## Statement

The content and views expressed in this article are those of the authors and do not necessarily reflect those of the Government of Canada.
